# Amblyopic astigmatism characteristics and surgical outcomes in younger children with severe congenital ptosis after frontalis suspension surgery

**DOI:** 10.1186/s12886-023-02804-9

**Published:** 2023-02-07

**Authors:** Yilan Tan, Xilang Wang, Jing Fu, Jing Tang, Jianbo Xiang, Lijuan Tao, Yulin Luo

**Affiliations:** Department of Ophthalmology, Hunan Children’s Hospital, No. 86 Ziyuan Road, Changsha City, Hunan Province China

**Keywords:** Congenital ptosis, Frontalis suspension, Astigmatism, Complication

## Abstract

**Background:**

To examine the astigmatism characteristics and surgical outcomes in patients with unilateral severe congenital ptosis following frontalis suspension surgery.

**Methods:**

We included 53 congenital ptosis patients who underwent frontalis suspension surgery in Hunan Children's Hospital. Each patient underwent a refractive examination before and after surgery to assess astigmatism. We also evaluated the effects and complications associated with the procedure.

**Results:**

Degree of astigmatism in ptotic and fellow eyes was − 1.45 ± 0.59 D and − 0.66 ± 0.51 D before surgery. Ratio of severe astigmatism in ptotic and fellow eyes was 51.3 and 12.8%. The fellow eyes presented with with-the-rule astigmatism (WR; 71.8%) and against-the-rule astigmatism (AR; 20.5%) types, with no cases of oblique astigmatism (OA). Ptotic eyes demonstrated higher frequencies of AR (59.0%) and OA (10.2%) than did fellow eyes. Furthermore, the former showed increased astigmatism, followed by a gradual decrease at the 6-month, before significantly decreasing at the 1-year postoperatively. The ratio of postoperative AR and OA astigmatism cases in ptotic eyes decreased to 35.9 and 7.7% 1 month postoperatively. However, there was a postoperative increase in the WR ratio from 30.8 to 56.4% after 1 month. Kaplan-Meier survival analysis showed a success rate of 81.4% at 6 months and 62.9% at 12 months which was influenced by the following complications: suture reaction, epithelial keratopathy, infection and granuloma, lid lag, and recurrence.

**Conclusion:**

Monocular congenital ptosis could develop severe astigmatism and higher frequency of AR or OA, early surgery may ameliorate astigmatic amblyopia.

## Backgroud

Congenital ptosis is a common condition characterized by drooping of the upper eyelid leading to visual, cosmetic, and psychological problems in children [1]. Correctional surgery is typically recommended for patients older than 3 years, to ensure adequate development of levator and frontalis muscle function, patient cooperation, and decreased anesthetic risks. However, it is imperative to consider visual acuity as a necessary parameter in children with a severe degree of unilateral ptosis. Therefore, the ideal time to conduct the procedure remains controversial [2, 3].

In our opinion, prevention of visual impairment should be the priority when treating severe unilateral congenital ptosis patients younger than 3 years, who are susceptible to amblyopia [4, 5]. Several surgeons have suggested an immediate surgical intervention to prioritize the development of visual acuity and binocular vision instead of improving the physical appearance of the patient [6]. Ptosis can, therefore, be addressed at a considerably early stage in the disease.

Here, we have reported regarding patients (age < 2 years) undergoing severe unilateral congenital ptosis correction using frontalis suspension with PTFE. We examined the refractive error characteristics, particularly the astigmatism status of the subjects with a detailed refractive examination before and after surgery. Furthermore, we also evaluated the effects and common complications associated with the procedure using the Kaplan-Meier survival analysis.

## Methods

This was a retrospective review that approved by the Ethics Committee of the Hunan Children’s Hospital (KS2015–48) and all subjects provided written informed consent.

We prospectively examined the medical records of all patients, younger than 2 years, who underwent frontalis suspension surgery with PTFE to address a severe unilateral congenital ptosis. Between January of 2016 and December of 2017, we recruited 53 Chinese patients with severe unilateral congenital ptosis, and excluded those presenting with ptosis secondary to systemic diseases or other eyelid diseases. Patient data including age, sex, time of operation, margin reflex distance, astigmatism degree and type measured with refractive examination under atropine was recorded pre- and postoperatively. Postoperative complications were also described. Surgical indications were unilateral congenital ptosis that was severe enough to cover the 50% of the pupil and possibly cause amblyopia. Each patient underwent follow-up examinations for more than 1 year after surgery, and were photographed regularly.

Routine ophthalmic examinations were performed in all patients. Levator and frontalis muscles function was unavailable because the patients were uncooperative. Cycloplegic refraction was measured after administrating 1% Atropine eye gel, thrice per day for 3 days. All procedures were performed using a handheld retinoscopy by optometrist preoperatively and 1 month, 6 months, and 1 year postoperatively. We used the minus cylinder to express all refraction measurements for consistency. We used the standard definition of astigmatism referred by Griepentrog [7]. Preoperative refraction was checked in all patients to compare the astigmatism status between ptotic and fellow eyes. During the follow-up, the patients also underwent a refractive examination to assess the differences of astigmatism in the ptotic and fellow eyes before and after surgery. Since the refractive data 1 year after the surgery of certain patients was missing due to an absence of follow-up data, we included and analyzed the refractive data of only 39 patients.

Each procedure was performed by two operators with general anesthesia. We used the frontalis suspension of the double rhomboid approach [8] with PTFE. Surgical success was defined by the presence of an acceptable eyelid position and height. Recurrence was defined as the eyelid covering the visual axis again after surgery. We documented the eyelid position using the photo taken at every follow-up. Kaplan-Meier survival analysis was performed with the failure time being measured as the time from initial surgery until disease recurrence. We also recorded the complications for each case.

Statistical analysis was performed using software Prism 5.0 (Graphpad Software, San Diego, CA, USA). Statistical significance was considered at *P* < 0.05. All data are expressed as the mean ± standard deviation. Paired t-test was used to compare the astigmatism between fellow and ptotic eyes before surgery. Chi-square test was used to compare the frequency of severe astigmatism (≤ − 1.50 DC) and astigmatism type between fellow and ptotic eyes before surgery. One-way ANOVA was used to compare the astigmatism degree before and after surgery in both ptotic and fellow eyes. Kaplan-Meier analysis was used to estimate surgical failure, defined as the eyelid covering the visual axis postoperatively.

## Results

We included 53 patients (34 boys, 19 girls) in the analysis; their ages ranged from 8 to 23 months at the time of surgery (mean age, 14.7 ± 5.6 months). The average follow-up time ranged from 6 to 53 months (mean age, 15.4 ± 7.6 months). Each patient underwent frontalis suspension with PTFE to repair severe unilateral congenital ptosis (22 left and 31 right eyes). Although most patients were followed-up postoperatively for minimum 6 months, some were not examined at 1 year postoperatively. Therefore, there were 39 patients with complete clinical data for statistics.

We compared the astigmatism value and axis between ptotic and fellow eyes preoperatively (Table 1). The preoperative refractive examination data of the patients revealed that the degree of astigmatism in ptotic eyes (− 1.45 ± 0.59 D) was higher than that in in fellow eyes (− 0.66 ± 0.51 D) (*p* < 0.001). Moreover, the ratio of severe astigmatism which was less than − 1.50 D of ptotic eyes and fellow eyes were 51.3 and 12.8% (*p* < 0.001), respectively. Therefore, ptopic eyes could develop a more severe form of astigmatism which may impair the patient’s visual acuity. We categorized astigmatism into WR (with the rule astigmatism), AR (against the rule astigmatism) and OA (oblique astigmatism). Fellow eyes presented with both WR (71.8%) and AR (20.5%) types of astigmatism, with no cases of OA. Ptotic eyes presented with a relatively higher frequencies of AR (59.0%) and OA (10.2%) types of astigmatism, which can severely impair vision, than the fellow eyes (*p* < 0.001).

**Table 1:** Comparison of astigmatism degree and type between ptotic eyes and fellow eyes before surgery (*n*=39)

	Ptotic eyes	Fellow eyes	*P* value
Total eyes	39	39	
**Astigmatism degree**			
Mean ± SD	−1.45 ± 0.59D	−0.66 ± 0.51D	
(range)	(−3.5 ~ −0.5)	(−1.75 ~ ~ 0)	< 0.001*
**Astigmatism severity**			
≤ −1.50 DC (Number,%)	20 (51.3%)	5 (12.8%)	
> −1.50 DC (Number,%)	19(48.7%)	34(87.2%)	< 0.001**
**Astigmatism type**			
AR (Number,%)	23 (59.0%)	8 (20.5%)	
WR (Number,%)	12 (30.8%)	28 (71.8%)	
OA (Number,%)	4 (10.2%)	0(0%)	
NA (Number,%)	0(0%)	3(7.7%)	< 0.001**

*AR* Against the rule astigmatism, *WR* With the rule astigmatism, *OA* Oblique astigmatism, *NA* None of astigmatism

*Paired t-test, *P* < 0.05 means statistically significant **Chi-Square test, *P* < 0.05 means statistically significant

**Fig. 1:**
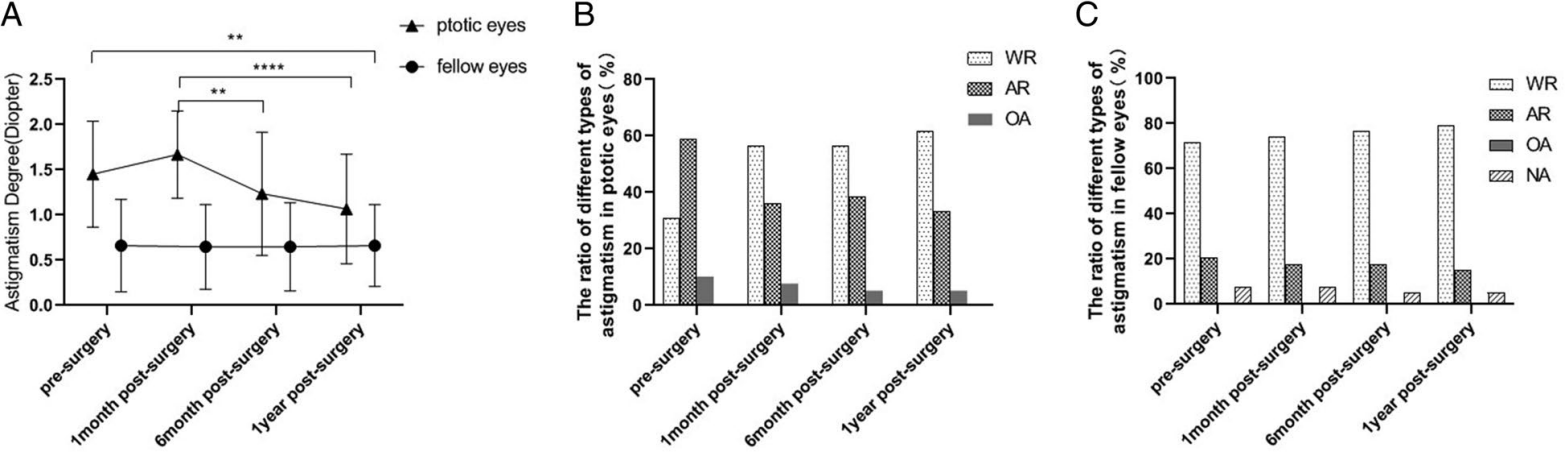
Comparison of astigmatism degree and type of ptotic eyes and fellow eyes pre-surgery, 1-month, 6-month, and 1-year post-surgery

Among the 53 patients, 39 underwent refractive examination 1-month, 6-month, and 1-year after operation. Since a number of patients were not present for the follow-up examinations, their refractive examination data was not included. The refractive data of 39 patients (Table 2), when compared with the patients’ preoperative values, the astigmatism degree of ptotic eyes had increased slightly 1-month after the surgery, and then decreased gradually during the follow-up at 6-month, and decreased significantly at 1-year after operation. There were no statistically significant differences between the preoperative, 1-month postoperative (*p* > 0.05), and the 6-month post-surgery (*p* > 0.05) data. However, the astigmatism value of ptotic eye decreased significantly between pre-surgery and 1-year post-surgery (*p* < 0.001, Fig. 1A). The ratio of AR and OA of ptotic eyes pre-surgery were 59.0 and 10.2% respectively (Table 3), which sharply decreased to 35.9 and 7.7% at 1-month post-surgery. However, the ratio of WR markedly increased from 30.8 to 56.4% at 1-month postoperatively. Difference of astigmatism types in the ptotic eyes were significant between the pre-surgery group and the 1-month post-surgery group (*p* < 0.05, Fig. 1B). Furthermore, there were no statistically significant differences between the data of the 1-month, 6-month, and 1-year post-surgery groups (*p* > 0.05, Fig. 1B). None of the fellow eyes showed any changes in astigmatism degree or type after 1-month, 6-month, and 1-year postoperatively than the preoperative measurements (*p* > 0.05, Fig. 1A, B, C).

**Table 2:** Comparison of astigmatism degree of ptotic eyes and fellow eyes pre-surgery, 1-month, 6-month, and 1-year post-surgery (Mean±SD, Diopter)

Follow-up Time		Astigmatism Degree	
	Ptotic eyes	(***n*** = 39)	Fellow eyes (***n*** = 39)
Pre-surgery	−1.45 ± 0.59		−0.66 ± 0.51
1-month	−1.69 ± 0.49		−0.65 ± 0.47
Post-surgery			
6-month	−1.26 ± 0.67		−0.65 ± 0.49
Post-surgery			
1-year post-surgery	−1.08 ± 0.59		−0.66 ± 0.45
F	7.89		0.0097
*P* value	< 0.0001*		0.998

*One-way ANOVA, *P* < 0.05 means statistically significant

**Table 3:** Comparison of astigmatism type of ptotic eyes and fellow eyes pre-surgery, 1-month, 6-month, and 1-year post-surgery

		Ptotic eyes (n = 39)			Fellow eye (n = 39)			
	AR	WR	OA	NA	AR	W	OA	N
						R		A
Pre-surgery	23	12	4	0	8	28	0	3
1-month	14	22	3	0	7	29	0	3
Post-surgery								
6-month	15	22	2	0	7	30	0	2
Post-surgery								
1-year post-surgery	13	24	2	0	6	31	0	2
_*×*_2		14.122				4.349		
*P* value		0.003*				0.411		

* Chi-Square test, *P* < 0.05 means statistically significant

*AR* Against the rule astigmatism, *WR* With the rule astigmatism, *OA* Oblique astigmatism, *NA* None of astigmatism

**Fig. 2:**
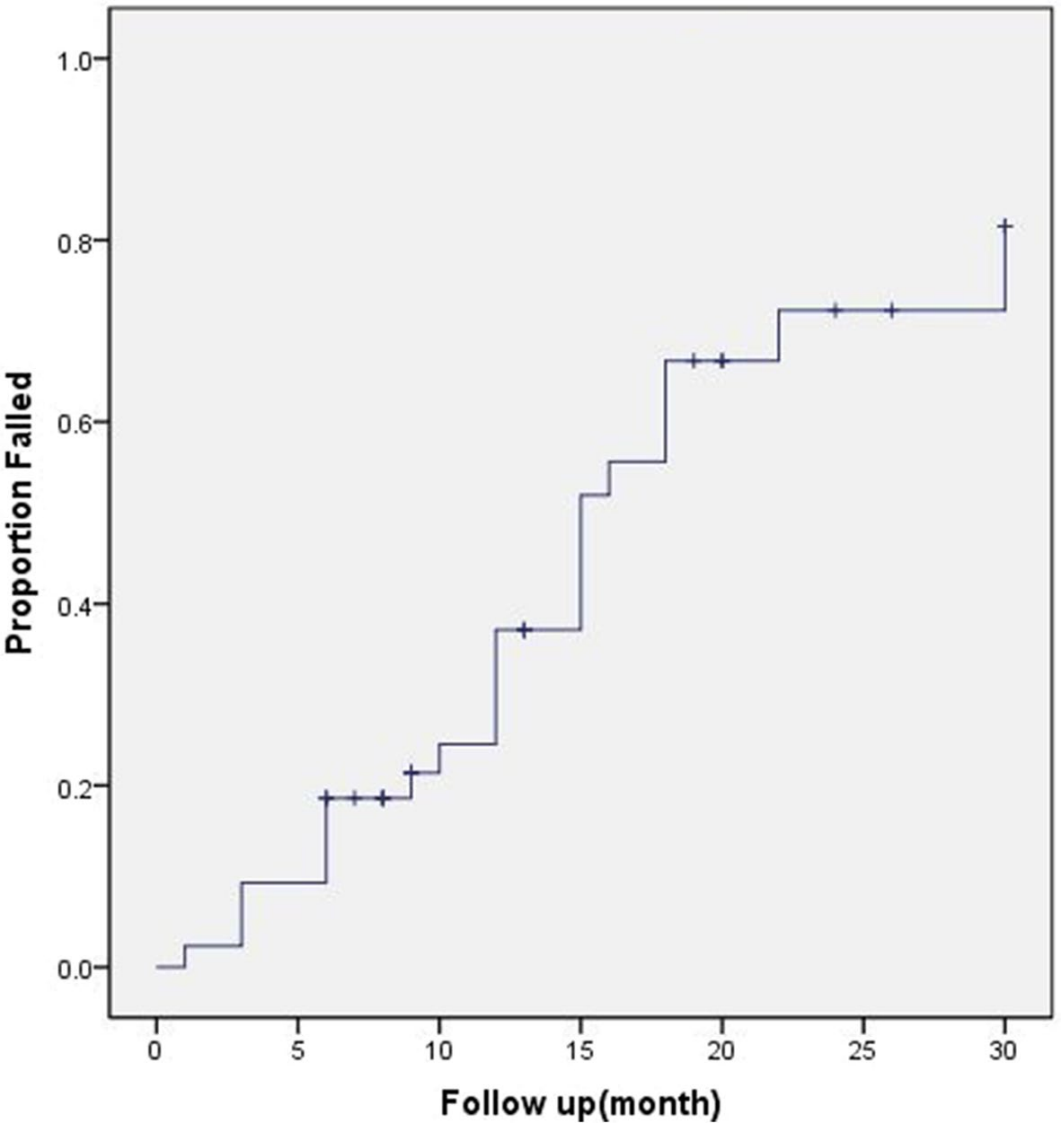
Kaplan-Meier survival analysis for the patients underwent frontalis suspension with Mersilk suture surgery for repairing severe unilateral congenital ptosis

**Fig. 3:**
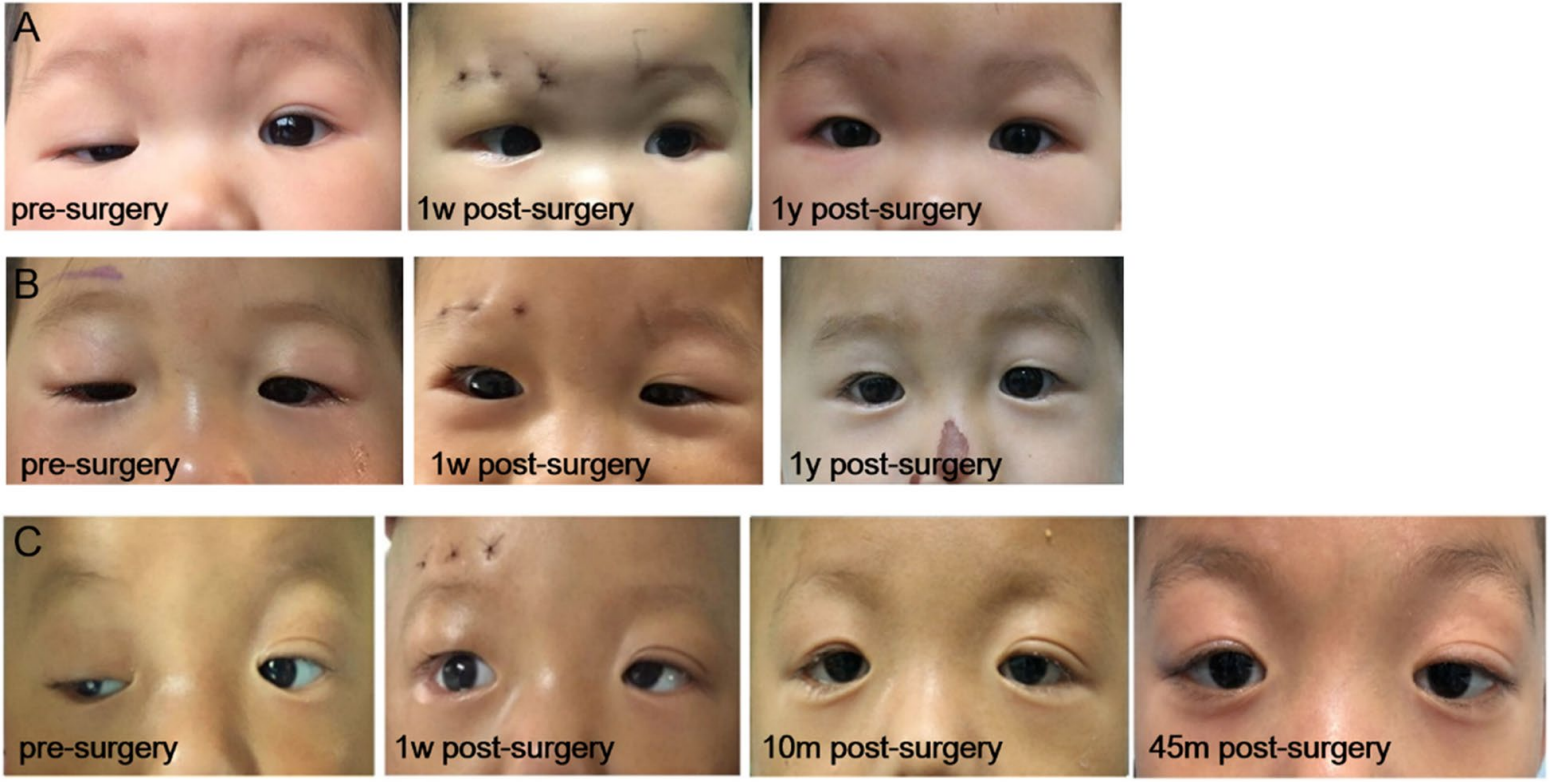
Appearance of patients pre-surgery and post-surgery

Kaplan-Meier survival analysis showed a success rate of 81.4% at 6 months, 62.9% at 12 months, 33.3% at 18 months, 27.7% at 22 months and 18.5% at 30 months (Fig. 2). After the 1-year follow up, most patients reported satisfactory surgical results (Fig. 3A, B). During the follow-up, the longest case with a successful surgical outcome was 45 months, which lasted from the initial surgery at the age of 12 months to the last visit at 4 year and 9 months (Fig. 3C). Until the last visit, 24 patients received the additional modified frontalis aponeurosis suspension surgery. All patients were older than 3 years when underwent the second surgery. For the remaining patients, additional procedures were needed for the obstructing of visual axis; however, their families declined for personal reasons.

Among the 53 patients, eight presented with early-stage complications 1 month after the surgery, which included suture reaction, epithelial keratopathy, infection, and granuloma formation. These complications did not affect the patients’ eyelid position, refraction or vision. Although lid lag could occur in nearly all patients postoperatively, we did not identify a severe case resulting in lagophthalmos and exposure keratopathy. The primary late-stage complication was recurrence. At the endpoint of follow-up, most of patients needed additional surgical intervention to recover the eyelid position.

## Discussion

Amblyopia in children with severe unilateral congenital ptosis primarily occurs from either an uncorrected refractive error such as the astigmatism, or form deprivation because of the droopy eyelid interfering with vision [9]. Corneal astigmatism is a major problem in congenital ptotic eyes, and cycloplegic refraction examination is a useful method to determine the astigmatism degree and type [10]. Our data supported the existing data by presenting a higher degree of astigmatism in the ptotic eye than that in the fellow eye before surgery. Meanwhile, the against-the-rule and oblique astigmatism ratio was also higher in the ptotic eye, which could lead to amblyopia in younger children. Our results were consistent with those reported by Paik, who also reported that the ptotic eyes had more severe astigmatism and a greater percentage of OA than the fellow eyes [11]. Both findings indicated that severe unilateral congenital ptosis could threaten children’s vision and possibly developed amblyopia. However, some parents and ophthalmologist only focused on the child’s appearance due to the drooping eyelids, and often ignored the visual damage caused by ptosis. Most children with unilateral ptosis could see with their normal eyes, due to which abnormal behaviors, including raising their eyebrow or the head-up position, were not often exhibited by them. Surgery should be considered to improve their appearance with age. However, the damage to vision or even amblyopia caused by ptotic eyelid could not be treated satisfactorily due to the reduced cortical plasticity in the older children. Additionally, it may irreversibly damage the visual function in children with ptosis. Based on the aforementioned results, pediatric ophthalmologists need to further reflect on the timing of surgery for children with severe unilateral congenital ptosis [12].

We aimed to avoid traditional approach suggesting that the ideal patient age to surgically treat congenital ptosis was minimum 3 years. We performed the frontalis suspension surgery withPTFE in patients (age < 2 years) to correct severe unilateral congenital ptosis. Here, we used a frontalis suspension technique as described previously [8], with a different suspension material. Frontalis suspension surgery is considered suitable to treat particularly young patients [13]. There were several different suspensory materials used in surgery [14]. Although the autogenous fascia lata is considered the best material for frontalis suspension, it has several associated surgical complications from the harvest site and possibility of permanent scar. Considering this, most surgeons believe that it should not be harvested in children under the age of 4 years due to the immaturity of the leg [15, 16]. Some alternative materials [17, 18] including banked fascia lata, Mersilene mesh and silicone rods are not commonly used in our hospital, Hunan province of China. Therefore, frontalis suspension using PTFE method was an appropriate corrective option for our patients. This surgical technique is considered easy to master, is minimally invasive, with inexpensive suspensory material, is available, an generates reproducible and excellent cosmetic and functional results during the short-term follow up.

According to the result of Kaplan-Meier survival analysis, 33 children demonstrated good surgical results at 12 months after surgery, with their binocular eyelid cleft symmetry and upper eyelids in an acceptable position and contour. During the follow-up, the parents were satisfied with their child’s appearance and improvements in psychological inferiority. However, only 10 children maintained their good results when followed-up at the 30-month time point postoperatively. In brief, most patients showed disease recurrence at the end of follow-up. Our surgical success rate was slightly lower than of Ho [19], which could be possible due to the following reasons: the suture was not fixed on the frontal muscle and tarsus firmly during the surgery; absence of scar adhesion between tissues postoperatively, thus the tarsus was cut by the suture and shifted due to the effect of eyelid gravity; and ametropia of the ptotic eye was not corrected in time after surgery. Since the patient only used his/her normal eye, ptosis recurred due to the poor fixation and vision of ptotic eye. In addition, the younger age and severity of the ptotic eye were also important reasons for recurrence. Additionally, we summarized the postoperative complications. At the early stage after surgery, only eight children developed mild complications including suture reaction, epithelial keratopathy, infection and granuloma formation. We treated them promptly to avoid any adverse effects on the cornea and visual function of the patients. Similar to other types of ptosis correction surgery, including the superior palpebral levator muscle resection, almost all patients developed lid lag during the early stage of surgery. This was the common problem of all ptosis correction surgeries. The severity of eyelid ptosis was proportional to the degree of lid lag. However, the eyelid lag could improve eventually, which reduced the risk of exposure keratitis. The results of our clinical study were consistent with that reported before [19, 20]. In summary, frontalis suspension surgery withPTFE was safe for treating severe unilateral congenital ptosis patients under 2 years of age. The subsequent effects were stable and satisfactory for at least 1 year postoperatively, which could help the patients’ smooth transition to the reoperation after the age of 3 years.

Whether the correction of ptotic eyelid by the surgery could reduce the damage of vision and incidence rate of amblyopia caused by severe ptosis remains unclear. Therefore, we comparatively analyzed the astigmatism degrees and types in the ptotic eye before and after surgery. According to our study, because of the removal of pressure and occlusion on the eyeball caused by the drooping eyelid, the astigmatism degree slightly improved 1-month postoperatively, and then decreased significantly since 6-month after surgery. Meanwhile, the type of astigmatism in the ptotic eye changed from AR and OA, which significantly damaged the vision in than WR, which had a considerably lower effect on vision since 1 month after the procedure. After 1-year follow up, the astigmatism degree of the ptotic eyes was significantly lower than that before surgery, similar to the proportion of AR and OA astigmatism of ptotic eye. The results of refraction follow-up suggested that, to a certain extent, the surgery could reduce visual damage and amblyopia incidence rate caused by severe ptosis during the critical period of visual development. Our clinical observations revealed that frontalis suspension surgery withPTFE was invaluable in treating severe unilateral congenital ptosis patients (age < 2 years). It should be noted that the astigmatism degree of ptotic eye improved slightly 1-month after surgery, which may have been related to edema of the upper eyelid and surrounding tissues at the very early stage after surgery [21, 22]. Furthermore, average patient age in our study was considerably small, thus they could not accurately follow instructions of the doctors, nurses, or even their parents to keep their eyelids bandaged, or remain still after surgery. All of these would aggravate the eyelid edema or even hematoma, which may compress the cornea and increase astigmatism preoperatively. Therefore, we should instruct the parents to take the patients for routine reexaminations including the refractive examination at the 1-month point postoperatively. Changes in refraction should be immediately addressed by replacing the glasses in time to ensure clear vision and reduce chances of amblyopia.

In conclusion, considering the relatively stable eyelid position, prophylaxis against severe amblyopia, inexpensive surgical procedure, and considerable improvements in the young patients after more than 1 year postoperatively, we recommend the frontalis suspension surgery withPTFE to treat unilateral severe congenital ptosis. Although the present study was prospective, there might be biases regarding the loss of follow-up data in several patients. The relatively short follow-up period was also another limitation. Since the patients were too young, they were not cooperative with a number of tests, therefore there were no data of their corneal topography, along with the fact that several refraction results are absent. A prospective, long-term and multicenter-based study is being planned. The finding of the present study suggested that more attention should be paid to early surgical correction of unilateral severe congenital ptosis to avoid the development of severe astigmatism and amblyopia.

## Data Availability

The datasets used and/or analysed during the current study are available from the corresponding author on reasonable request.

## Abbreviations

WR: With the rule astigmatism
AR: Against the rule astigmatism
OA: Oblique astigmatism
